# Inhibition of ERK1/2 Restores GSK3β Activity and Protein Synthesis Levels in a Model of Tuberous Sclerosis

**DOI:** 10.1038/s41598-017-04528-5

**Published:** 2017-06-23

**Authors:** Rituraj Pal, Vitaliy V. Bondar, Carolyn J. Adamski, George G. Rodney, Marco Sardiello

**Affiliations:** 1 Department of Molecular Physiology and Biophysics, Baylor College of Medicine, One Baylor Plaza, Houston, TX 77030 USA; 2 Department of Molecular and Human Genetics, Baylor College of Medicine, One Baylor Plaza, Houston, TX 77030 USA; 3 Jan and Dan Duncan Neurological Research Institute, Texas Children’s Hospital, Houston, TX 77030 USA; 4 Cardiovascular Research Institute, Baylor College of Medicine, One Baylor Plaza, Houston, TX 77030 USA; 5 Center for Space Medicine, Baylor College of Medicine Bioscience Research Collaborative, Houston, TX 77030 USA

## Abstract

Tuberous sclerosis (TS) is a multi-organ autosomal dominant disorder that is best characterized by neurodevelopmental deficits and the presence of benign tumors. TS pathology is caused by mutations in tuberous sclerosis complex (*TSC*) genes and is associated with insulin resistance, decreased glycogen synthase kinase 3β (GSK3β) activity, activation of the mammalian target of rapamycin complex 1 (mTORC1), and subsequent increase in protein synthesis. Here, we show that extracellular signal–regulated kinases (ERK1/2) respond to insulin stimulation and integrate insulin signaling to phosphorylate and thus inactivate GSK3β, resulting in increased protein synthesis that is independent of Akt/mTORC1 activity. Inhibition of ERK1/2 in *Tsc2* ^−/−^ cells—a model of TS—rescues GSK3β activity and protein synthesis levels, thus highlighting ERK1/2 as a potential therapeutic target for the treatment of TS.

## Introduction

Tuberous sclerosis (TS), a hereditary autosomal syndrome caused by defects in either *TSC1* or *TSC2* genes, is characterized by a wide spectrum of clinical manifestations in multiple organs^1–3^. Molecular pathology of TS includes resistance to insulin signaling, hyper-phosphorylation and thus inactivation of GSK3β, aberrant activation of mTORC1, and elevated levels of protein synthesis^4, 5^. TSC1 and TSC2 form a heterodimeric tumor suppressor complex that negatively regulates the activity of mTORC1, a central hub for the regulation of protein synthesis, cell growth and proliferation^2, 6^. Several upstream signaling pathways that sense growth factors and energy levels converge to TSC2 and determine its activation status, thus resulting in the modulation of the activity of mTORC1 and thereby of the rate of protein synthesis^7^. Loss of TSC1 or TSC2 protein indeed results in constitutively active mTORC1 and elevated levels of protein synthesis^5^.

mTORC1 is a highly conserved regulator of transcription, ribosomal biogenesis, translation and cell growth and is one of the most highly integrated signaling nodes present in all cell types^8, 9^. In response to amino acids, mTORC1 is recruited to lysosomes where it is fully activated by its potent and essential direct activator, Rheb guanosine triphosphatase (Rheb GTPase)^10^. Upon insulin stimulation, PI3K activates protein kinase B (Akt/PKB), which then directly phosphorylates TSC2 resulting in the dissociation of the TSC1/2 complex from lysosomes where mTORC1 activation takes place^2^. Because TSC2 acts as a GTPase-activating protein (GAP) specific for Rheb, Akt inhibition of TSC2 promotes Rheb-GTP-dependent mTORC1 activation^11^. In cells lacking TSC2, mTORC1 and its substrates remain constitutively active and insensitive to insulin availability, which, in turn, results in hyperactivation of the translational machinery.

Glycogen synthase kinase 3 (GSK3) is implicated in multiple biological processes including regulation of protein synthesis, cell proliferation and survival^12–14^ and is found to be phosphorylated and inactivated in multiple cancer types^15, 16^. GSK3 activity is inhibited through phosphorylation of serine 21 in GSK3α and serine 9 in GSK3β^17^. Upon insulin stimulation, Akt-mediated phosphorylation of GSK3β inhibits GSK3β activity and causes its substrate, eukaryotic initiation factor 2B (eIF2B), to become dephosphorylated and thus activated^17–19^. Insulin-dependent activation of GSK3β-eIF2B pathway therefore stimulates activation of protein synthesis^20^. Recent studies have shown that GSK3 phosphorylation is also sensitive to mTORC1 activity^4, 21^. In cells lacking *TSC2*, where mTORC1 remains constitutively active and resistant to insulin stimulation, GSK3β is phosphorylated by S6K1, a direct downstream substrate of mTORC1^4^.

Extracellular signal–regulated kinases (ERK1/2) have long been implicated in protein synthesis^22, 23^. The growth factors, EGF and IGF1, stimulate a kinase cascade leading to the activation of ERK1/2 and Ras, which then transmit the upstream signals to TSC1/2 by specific phosphorylation events^24, 25^ to activate mTORC1. Previous studies have shown that ERK1/2 integrates IGF1 signal to phosphorylate and inactivate GSKβ in an Akt-independent manner^26, 27^. Together, these findings suggest that ERK1/2 could possibly play a significant role in insulin regulation of GSKβ activity and protein synthesis in TS. In this study, we show that insulin activation of ERK1/2 is necessary for insulin-mediated phosphorylation and inactivation of GSK3β. We demonstrate that insulin-mediated activation of ERK1/2 results in phosphorylation of GSK3β at S9 independently of Akt/mTORC1 activity in *Tsc2* null mouse embryonic fibroblasts. In addition, we show that inhibition of ERK1/2 rescues GSK3β activity and restores protein synthesis in *Tsc2* ^−/−^ MEFs to normal levels. Together, these findings highlight ERK1/2 as a potential therapeutic target for the treatment of tuberous sclerosis.

## Results

### Insulin regulation of ERK1/2 is independent of Akt/mTORC1 signaling

**Figure 1:**
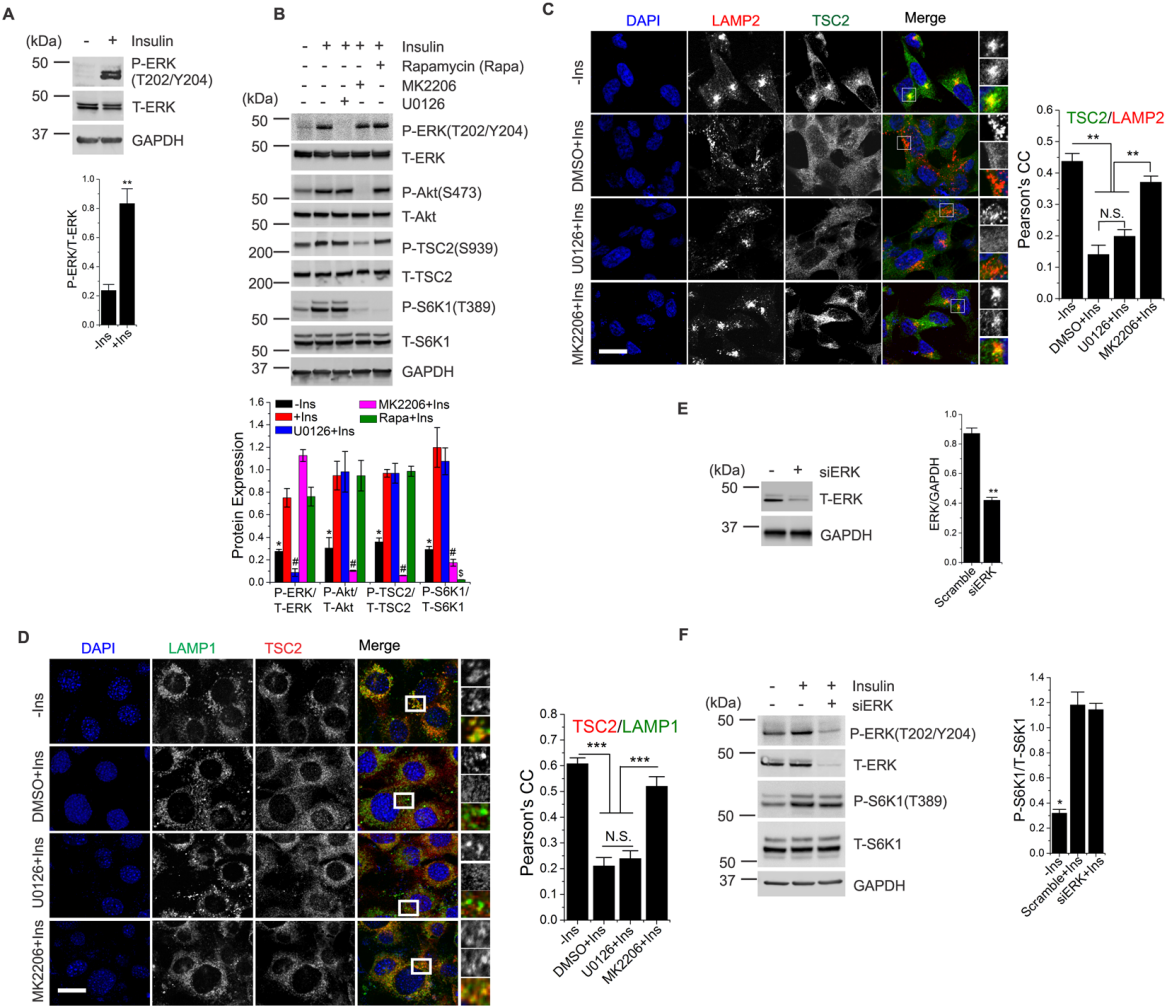
Insulin regulation of ERK and Akt/mTORC1 are independent of each other. (**A**) HEK cells were starved of serum (16 h) prior to insulin stimulation (1 µM, 15 min). Cell lysates were probed with antibodies as indicated. Quantification of three independent experiments is reported in the bar diagrams. (**B**) HEK cells were starved of serum (16 h) and treated with indicated drugs for 2 h prior to insulin stimulation (1 µM, 15 min). Lysates were analyzed by immunoblot assay using antibodies as indicated. Quantification of three independent experiments is reported in the bar diagrams. *, ^#^ and ^$^ indicate significant differences between all conditions in each group. (**C**) HEK cells were treated as in *B* prior to immunofluorescent labeling of endogenous LAMP2 (red) and TSC2 (green). Bar indicates 30 μm. (**D**) *Tsc2* ^+/+^ MEFs were treated as in *B* prior to immunofluorescent labeling of endogenous LAMP1 (green) and TSC2 (red). Bar indicates 20 μm. Representative cells are shown in *C* and *D*, where yellow or orange pixels indicate co-localization in the merged images. Zoomed images represent the area under the white box. Quantification of three independent experiments and at least 30 cells is reported in the bar diagrams in *C* and *D*. (**E**) HEK cells were transfected with siRNA targeted to *ERK* (or scrambled controls) for 72 h prior to cell lysis. Lysates were probed with antibodies as indicated. (**F**) HEK cells transfected with *siERK* or scramble for 56 h prior to 16 h-serum starvation followed by insulin stimulation (1 µM, 15 min) where indicated. Lysates were probed with antibodies as indicated. Quantification of three independent experiments is reported in the bar diagrams in *E* and *F*. All quantitative analyses are reported as mean ± SEM with a significance level of *p < 0.05, **p < 0.01, ***p < 0.001, ^#^ *p* < 0.05 and ^$^ *p* < 0.01. N.S. indicates not significant. GAPDH was used as a loading control in all immunoblot assays. Cropped blots from full-length gels are displayed in *A*, *B*, *E* and *F*.

TS pathology is characterized by constitutively active mTORC1 that is insensitive to insulin availability. To test the effects of the manipulation of ERK activity in a model of TS, we first investigated the interplay between ERK and insulin regulation of Akt-mTORC1 signaling. Time- and dose-dependency studies have previously established that an optimal activation of the Akt/mTORC1 pathway in human cell lines or mouse embryonic fibroblasts (MEFs) can be obtained by stimulating serum-depleted cells with 1 µM insulin for 15 min^2^. Using similar conditions, we found that insulin activates ERK1/2 (hereafter referred as ERK) activity in HEK-293 cells (hereafter referred as HEK) (Fig. 1A). Consistent with previous studies^2^, we found that a 15-min period of insulin stimulation resulted in a significant increase in phosphorylation of Akt and the Akt TSC2 target residue S939 (Fig. 1B). Accordingly, robust activation of mTORC1 was observed in these conditions. Pharmacological inhibition of ERK with U0126^28, 29^ selectively suppressed the insulin-mediated activation of ERK without altering the phosphorylation levels of Akt, TSC2 or S6K1 (Fig. 1B). As expected, inhibition of Akt by MK2206^2^ or of mTORC1 by rapamycin (Rapa)^30^ abolished insulin-mediated activation of mTORC1. Insulin stimulates mTORC1 activity by promoting the dissociation of TSC2 from lysosomes *via* Akt-mediated phosphorylation of TSC2^2^. A previous study reported that ERK also phosphorylates TSC2, but at an amino acid residue that is different from the Akt target sites^25^. To test whether insulin-mediated activation of ERK controls spatial localization of TSC2, we performed confocal microscopy analysis of HEK cells (Fig. 1C) and MEFs (Fig. 1D) using antibodies specific to TSC2 and the lysosomal markers, LAMP1 and LAMP2. Consistent with previous studies, we observed that TSC2 was predominantly localized at lysosomes in serum-starved cells^2, 10^, which was significantly reduced upon insulin stimulation. Notably, inhibition of ERK by U0126 was unable to prevent insulin-induced dissociation of TSC2 from lysosomes (Fig. 1C,D). As a control, inhibition of Akt significantly prevented insulin-induced dissociation of TSC2 from lysosomes. Genetic inhibition of ERK using small interfering RNAs (Fig. 1E) significantly decreased the levels of total and phospho-ERK but, similar to ERK pharmacological inhibition, resulted in no changes in insulin-stimulated mTORC1 activity (Fig. 1F). Together, these results demonstrate that insulin regulation of ERK is independent of Akt/mTORC1 activity and that ERK activation does not alter Akt/mTORC1signaling.

### ERK1/2 regulate insulin-dependent protein synthesis independently of Akt/mTORC1 signaling

**Figure 2:**
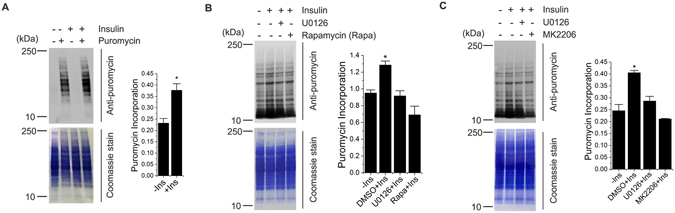
ERK regulates insulin-stimulated protein synthesis. (**A**) HEK cells were starved of serum (16 h) prior to insulin stimulation (1 µM, 15 min). Cells were then incubated with puromycin for 30 min followed by cell lysis. Lysates were probed with an antibody to puromycin. Blot was stained with coomassie at the end of the immunoblot assay. (**B** and **C**) HEK cells were starved of serum (16 h) and treated with indicated drugs for 2 h prior to insulin stimulation (1 µM, 15 min). Cells were then incubated with puromycin for 30 min followed by cell lysis. Lysates were probed with an antibody to puromycin. Blot was stained with coomassie at the end of the immunoblot assay. Quantifications of three independent experiments are reported in the bar diagrams in *A*, *B* and *C*. All quantitative analyses are reported as mean ± SEM with a significance level of *p < 0.05. *Indicates significant differences between all conditions in *B* and *C*.

TS pathology is associated with constitutively active Akt-mTORC1 signaling pathway that induces elevated levels of protein synthesis. We tested whether ERK regulates protein synthesis in an Akt-mTORC1 independent manner. To test this hypothesis, we used SUnSET, a nonradioactive puromycin end-labeling assay^31–34^. Immunoblot analysis in HEK cells stimulated with insulin, which activates Akt-mTORC1 signaling, showed a significant increase in protein synthesis compared to starved cells (Fig. 2A). Importantly, the insulin-dependent increase in protein synthesis was significantly diminished by ERK inhibition, similar to control experiments conducted by inhibiting Akt (Fig. 2B) or mTORC1 (Fig. 2C) activities. Together with our findings that insulin-mediated activations of ERK and Akt/mTORC1 are independent of each other, these results indicate that ERK regulates insulin-mediated protein synthesis in an Akt/mTORC1-independent manner.

### Insulin regulates ERK1/2 activity in *Tsc2*^−/−^ cells

**Figure 3:**
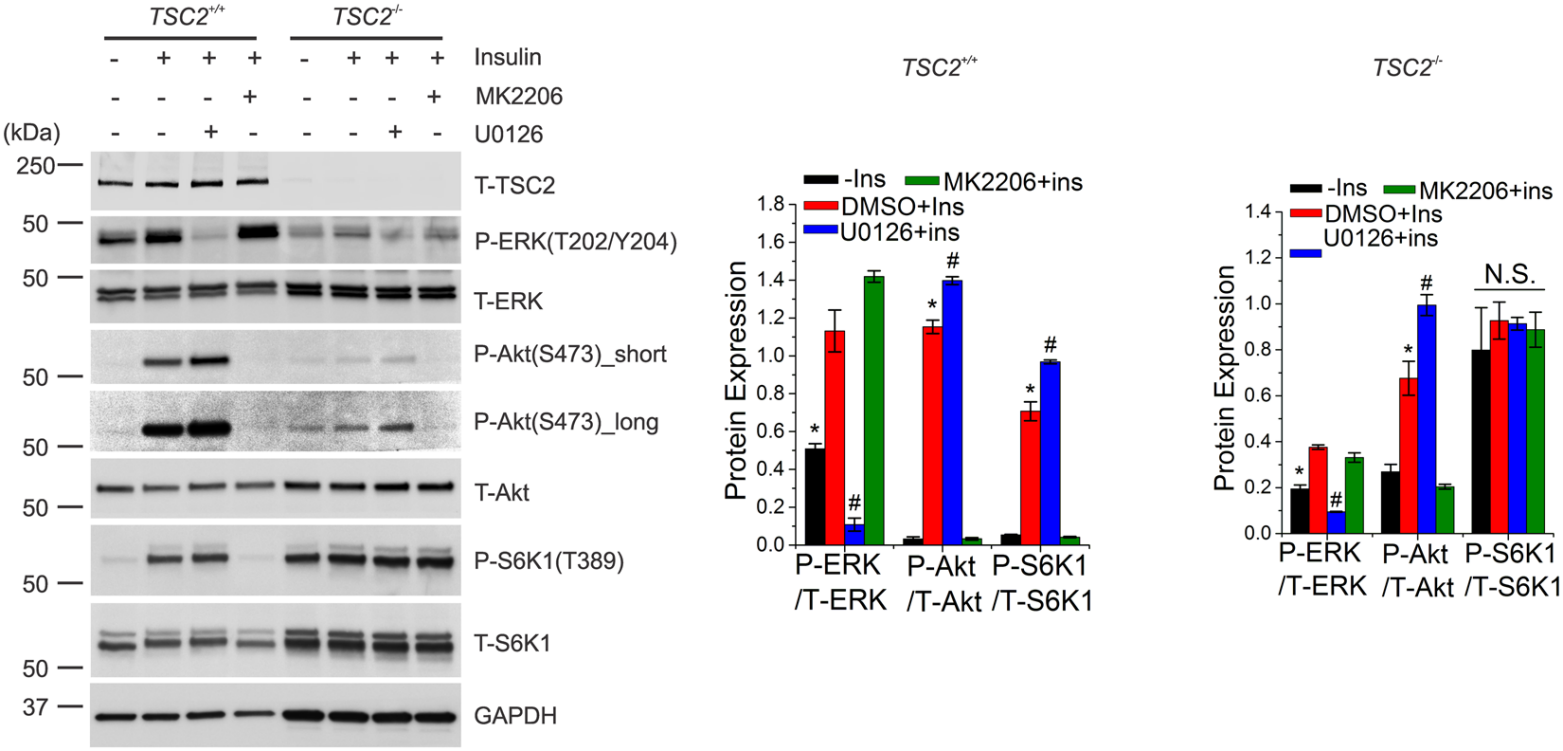
Insulin regulates ERK activity in both *Tsc2* ^+/+^ and *Tsc2* ^−/−^ cells. *Tsc2* ^+/+^ and *Tsc2* ^−/−^ MEFs were starved of serum (16 h) and treated with the indicated drugs for 2 h prior to insulin stimulation (1 µM, 15 min). Lysates were analyzed by immunoblot assay using antibodies as indicated. GAPDH was used as a loading control in all immunoblot assays. Quantification of three independent experiments is reported in the bar diagrams. All quantitative analyses are reported as mean ± SEM with a significance level of *p < 0.05 and ^#^ *p* < 0.05. N.S. indicates not significant. * and ^#^indicate significant differences between all conditions in each group. Cropped blots from full-length gels are displayed.

Based on these results, we hypothesized that ERK activity remains sensitive to insulin in TS. To test this hypothesis, we used *Tsc2* ^−/−^ MEFs, where mTORC1 is constitutively active and resistant to insulin. In wild-type MEFs (*Tsc2* ^+/+^), insulin significantly activated ERK, Akt and mTORC1, and neither Akt nor mTORC1 was suppressed by inhibition of ERK (Fig. 3). In these cells, insulin-mediated activation of Akt-mTORC1 was abolished upon Akt inhibition. In *Tsc2* ^−/−^ MEFs, the activity of mTORC1 was insensitive to inhibition of either ERK or Akt (Fig. 3), however, insulin stimulation was able to activate ERK. Consistent with earlier observations indicating that mTORC1 shows feedback inhibition of PI3K-Akt pathway in *Tsc2* ^−/−^ cells^4^, we also found that Akt activity was decreased in *Tsc2* ^−/−^ cells compared to *Tsc2* ^+/+^ cells. Interestingly, a similar decrease was also observed for ERK activity, suggesting that mTORC1 could possibly show feedback inhibition of ERK in conditions of insensitivity to insulin such as the absence of TSC2. Collectively, these results indicate that insulin is able to regulate ERK activity in a model of TS.

### ERK1/2 mediates insulin regulation of GSK3β activity in an Akt/mTORC1-independent manner

**Figure 4:**
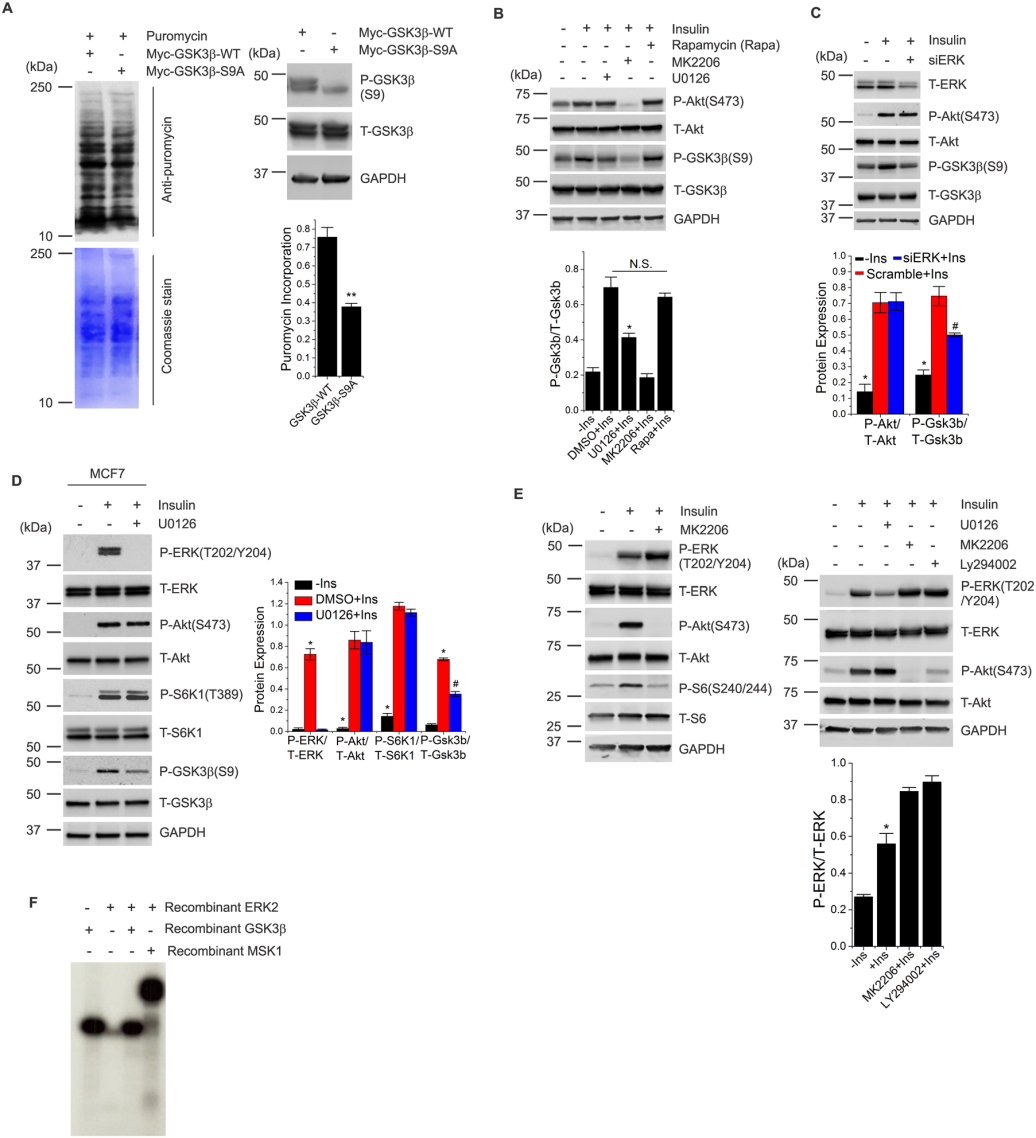
ERK integrates insulin signaling to GSK3β in an Akt-independent manner. (**A**) HEK cells were transfected with Myc-GSK3β-WT or Myc-GSK3β-S9A for 48 h prior to the addition of puromycin in the culture medium. Cell lysates were probed with an antibody to puromycin. The blot was stained with coomassie at the end of the immunoblot assay. Another set of cells were transfected with Myc-GSK3β-WT or Myc-GSK3β-S9A for 48 h prior to cell lysis. Lysates were probed with antibodies as indicated. (**B**) HEK cells were starved of serum (16 h) and treated with the indicated drugs for 2 h prior to insulin stimulation (1 µM, 15 min). Lysates were analyzed by immunoblot assay using antibodies as indicated. Quantification of three independent experiments is reported in the bar diagrams. * indicates significant differences between all conditions in each group. (**C**) HEK cells transfected with *siERK* or scramble for 56 h prior to 16 h-serum starvation followed by insulin stimulation (1 µM, 15 min) where indicated. Lysates were probed with antibodies as indicated. Quantification of three independent experiments is reported in the bar diagrams. * and ^#^ indicate significant differences between all conditions in each group. (**D**) MCF7 cells were treated as in *B* prior to the cell lysis. Cell lysates were analyzed with antibodies as indicated. Quantification of three independent experiments is reported in the bar diagrams. * and ^#^ indicate significant differences between all conditions in each group. (**E**) HEK cells were starved of serum and treated with the indicated drugs for 2 h prior to insulin stimulation. Lysates were analyzed by immunoblot assay using antibodies as indicated. (**F**) *In vitro* kinase assay (see method for complete details). Quantification of three independent experiments is reported in the bar diagrams. * Indicates significant differences between all conditions in each group. GAPDH was used as a loading control in all immunoblot assays. All quantitative analyses are reported as mean ± SEM with a significance level of *p < 0.05, **p < 0.01 and ^#^ *p* < 0.05. N.S. indicates not significant. Cropped blots from full-length gels are displayed in immunoblots.

GSK3β is a key regulator that modulates global protein synthesis by regulating components of eukaryotic translation initiation^20^. Upon insulin stimulation, Akt-mediated phosphorylation and suppression of GSK3β activity^17^ results in dephosphorylation and subsequent activation of the GSK3β substrate, eukaryotic initiation factor 2B (eIF2B)^17, 18^. Active eIF2B promotes protein synthesis by stimulating translation initiation^20, 35^. Our data indicate that ERK regulates insulin-induced protein synthesis in an mTORC1-independent manner. We then investigated whether ERK regulates protein synthesis in *Tsc2* ^−/−^ cells through inactivation of GSK3β. To test this possibility, we first transfected HEK cells with myc-tagged wild-type GSK3 (Myc-GSK3β-WT) or constitutively active GSK3 (Myc-GSK3β-S9A) constructs^36^. Immunoblot analysis of cells transfected with constitutively active GSK3β showed a significant decrease in protein synthesis (Fig. 4A). We next examined whether ERK regulates insulin-mediated phosphorylation and inactivation of GSK3β in an Akt/mTORC1 independent manner. Our results showed that insulin-induced phosphorylation of GSK3β was significantly reduced upon ERK inhibition by U0126 (Fig. 4B); however, no change in Akt activity was observed. As expected, inhibition of Akt also suppressed the phosphorylation of GSK3β. Notably, rapamycin did not alter the phosphorylation of GSK3β (Fig. 4B). siRNA-mediated silencing of *ERK* significantly decreased insulin-mediated phosphorylation of ERK and GSK3β, but again no obvious change was observed in Akt activity (Fig. 4C). To test that insulin regulation of ERK-GSK3β signaling is not cell-type specific, we used the MCF7 breast cancer cell line^37^. We found that insulin stimulation rapidly phosphorylates ERK, Akt, S6K1 and GSK3β. Insulin-mediated phosphorylation of ERK and GSK3β was significantly decreased by ERK inhibition; however, no significant changes were observed in Akt or mTORC1 activity (Fig. 4D). We then tested whether PI3K/Akt acts as an upstream regulator of ERK in response to insulin. Immunoblot analysis of HEK cells showed that treatment with an Akt inhibitor (MK2206) or a PI3K inhibitor (LY294002) did not decrease ERK phosphorylation but rather showed a slight, but significant, increase in ERK phosphorylation (Fig. 4E), indicating that Akt and ERK could possibly share a negative feedback loop. Together, these results demonstrate that ERK regulates insulin-mediated GSK3β activity independent of Akt/mTORC1. We also tested whether ERK directly phosphorylates GSK3β by performing *in vitro* kinase assay by using recombinant ERK2 and GSK3β proteins. We did not observe any obvious changes in GSK3β phospho-signal in the presence or absence of active ERK, differently from the known ERK substrate, MSK1^38^, that we used as a positive control (Fig. 4F). These results suggest that ERK does not directly phosphorylate GSK3β. A co-IP assay performed by pulling down endogenous ERK did not reveal any detectable interaction with GSK3β (data not shown). Future studies might establish so far unidentified ERK regulatory signals to the GSK3β that integrates insulin signaling to protein synthesis.

### ERK1/2 regulates GSK3β activity and protein synthesis in *Tsc2*^−/−^ cells

**Figure 5:**
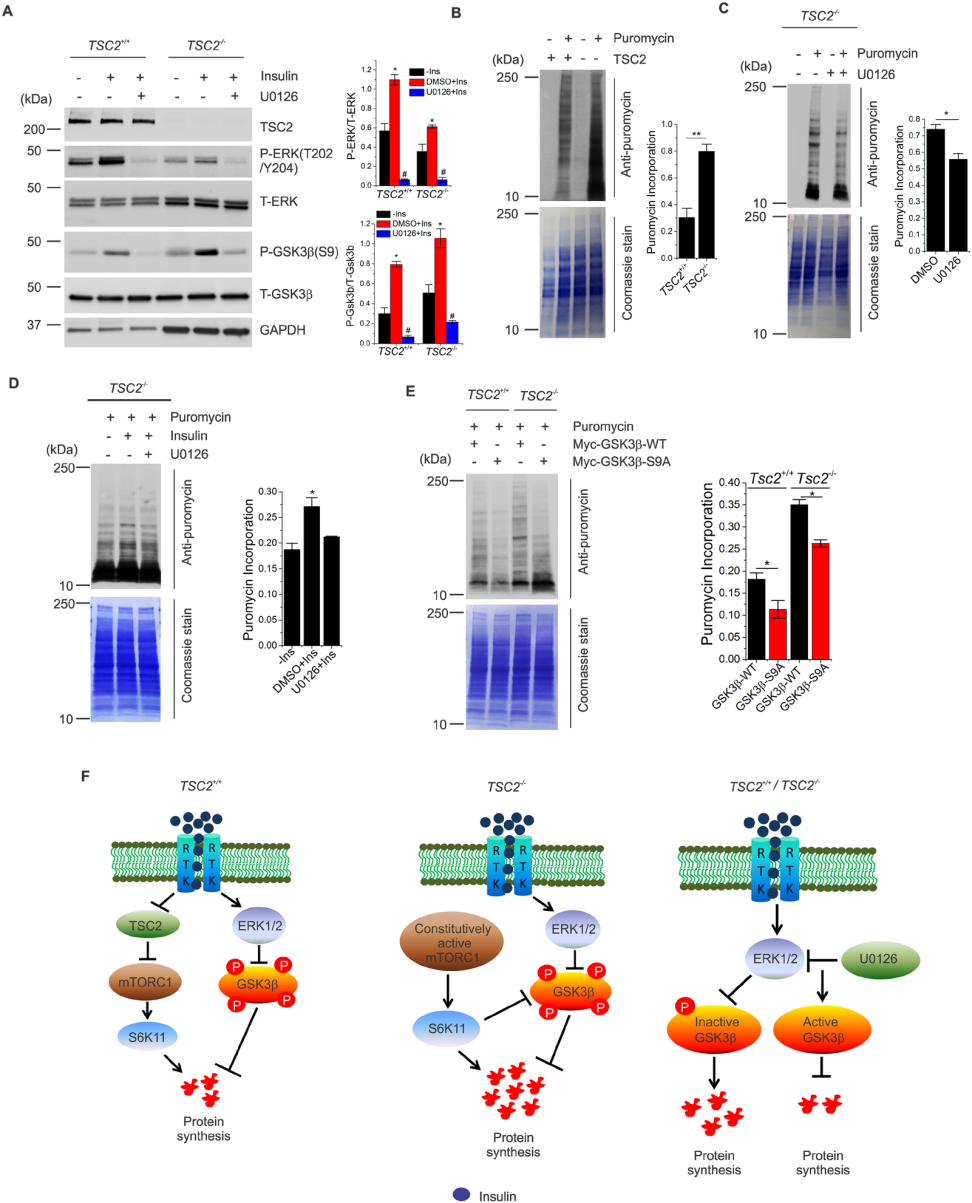
ERK restores GSK3β activity and protein synthesis levels in *Tsc2* ^−/−^ cells. (**A**) *Tsc2* ^+/+^ and *Tsc2* ^−/−^ MEFs were starved of serum (16 h) and treated with U0126 for 2 h prior to insulin stimulation (1 µM, 15 min). Lysates were analyzed by immunoblot assay using antibodies as indicated. GAPDH was used as a loading control in all immunoblot assays. Quantification of three independent experiments is reported in the bar diagrams. * and ^#^ indicate significant differences between all conditions in each group. (**B**) *Tsc2* ^+/+^ and *Tsc2* ^−/−^ MEFs were grown 24 h followed by treatment with puromycin for 30 min prior to cell lysis. (**C**) *Tsc2* ^−/−^ MEFs were grown 24 h followed by treatment with U0126 for 24 h prior to the addition of puromycin (30 min) in media. Cell lysates from *B* and *C* were probed with an antibody to puromycin. The blots were stained with coomassie at the end of the immunoblot assay. Quantification of three independent experiments is reported in the bar diagrams in *B* and *C*. (**D**) *Tsc2* ^−/−^ MEFs were starved of serum (16 h) and treated with U0126 for 2 h prior to insulin stimulation (1 µM, 15 min). Cells were then incubated with puromycin for 30 min followed by cell lysis. Lysates were probed with an antibody to puromycin. Blot was stained with coomassie at the end of the immunoblot assay. Quantifications of three independent experiments are reported in the bar diagrams. (**E**) *Tsc2* ^+/+^ and *Tsc2* ^−/−^ MEFs were transfected with Myc-GSK3β-WT or Myc-GSK3β-S9A for 48 h prior to the addition of puromycin in the culture medium. Cell lysates were probed with an antibody to puromycin. The blot was stained with coomassie at the end of the immunoblot assay. Quantitative analyses are reported as mean ± SEM with a significance level of *p < 0.05 and **p < 0.01 in *B*, *C*, *D* and *E*. (**F**) Model showing that *Tsc2* ^−/−^ MEFs have elevated level of protein synthesis compared to *Tsc2* ^+/+^ MEFs of protein synthesis. ERK integrates insulin signaling to GSK3β and protein synthesis in both *Tsc2* ^+/+^ and *Tsc2* ^−/−^ cells. Inhibition of ERK activates GSK3β and decreases protein synthesis level. Cropped blots from full-length gels are displayed in *A*.

Finally, we tested whether ERK regulates GSK3β activity and protein synthesis level in *Tsc2* ^−/−^ cells. Consistent with previous findings, we found an increased level of phosphorylated and inactivated GSK3β in *Tsc2* ^−/−^ cells compared to *Tsc2* ^+/+^ cells, which was further elevated in response to insulin stimulation (Fig. 5A). Similar to HEK and MCF7 cells, we found that insulin-mediated phosphorylation of ERK and GSK3β was suppressed by ERK inhibition in both *Tsc2* ^+/+^ and *Tsc2* ^−/−^ cells (Fig. 5A), confirming that ERK regulation of GSK3β activity in response to insulin stimulation is active in TS. We then investigated whether ERK regulates protein synthesis in *Tsc2* ^−/−^ MEFs. As expected, *Tsc2* ^−/−^ cells showed a significant increase in the level of protein synthesis compared to *Tsc2* ^+/+^ cells (Fig. 5B), which was significantly decreased upon ERK inhibition (Fig. 5C). Immunoblot analysis of *Tsc2* ^−/−^ MEFs stimulated with insulin showed a significant increase in protein synthesis compared to starved cells (Fig. 5D). Importantly, the insulin-dependent increase in protein synthesis was significantly diminished by ERK inhibition. We then investigated whether GSK3β modulates protein synthesis in *Tsc2* ^−/−^ MEFs. To test this possibility, we transfected *Tsc2* ^−/−^ MEFs with myc-tagged wild-type (Myc-GSK3β-WT) or constitutively active (Myc-GSK3β-S9A) GSK3 constructs. Immunoblot analysis of cells transfected with constitutively active GSK3β showed a significant decrease in protein synthesis in both *Tsc2* ^+/+^ and *Tsc2* ^−/−^ cells (Fig. 5E). Collectively, these results demonstrate that inhibition of ERK rescues GSK3β activity and restores the levels of protein synthesis in conditions of absence of TSC2 and hyperactivation of mTORC1 (Fig. 5F).

## Discussion

Nutrient availability controls the efficient transition between anabolic and catabolic states in most organisms^8^. Several components of the cell regulate cell growth by coordinating upstream signals from growth factors and sensors of intracellular energy levels and amino acid availability, which are often deregulated in cancer, diabetes and other metabolic disorders^8, 9^. Insulin, a major growth factor, plays a pivotal role in maintaining the metabolic homeostasis in many cell types^2^. Insulin signal funnels through the PI3K/Akt pathway to regulate two major regulators of cell metabolism, mTORC1 and GSK3, which maintain cellular homeostasis through protein and glycogen synthesis, respectively^17, 20^. Tuberous sclerosis complex (TSC) is a rare genetic disorder that is characterized by an increase in protein synthesis and a decrease in GSK3β activity. In this study, we find that ERK integrates insulin signaling to GSK3β to regulate global protein synthesis in *Tsc2* ^−/−^ cells, a model of tuberous sclerosis^39^. Inhibition of ERK restores both GSK3β activity and levels of protein synthesis in *Tsc2* ^−/−^ cells, thus identifying ERK as a candidate therapeutic target for the treatment of tuberous sclerosis. In addition, the finding that ERK regulates protein synthesis *via* modulation of GSK3β activity in an Akt/mTORC1-independent manner provides a new regulatory pathway that could be exploited for combinatorial therapeutic interventions in tuberous sclerosis.

Previous studies have shown that, in absence of TSC2, cells become resistant to insulin stimulation and GSK3β phosphorylation remains elevated^4^. Lack of TSC2 results in a constitutively active mTORC1, which in turn phosphorylates and inactivates GSK3β through hyperactivation of the mTORC1 substrate, S6K1^4^. mTORC1 regulation of GSK3 has gained significant interest over the past few years, as this mechanism could have implications in several pathological conditions such as cancer and diabetes, as well as tuberous sclerosis and lymphangioleiomyomatosis. Our finding that pharmacological inhibition of ERK inhibits GSK3β activity in *Tsc2* ^−/−^ cells suggests that modulation of ERK may be leveraged to regulate GSK3β activity in multiple pathological conditions characterized by mTORC1 hyperactivation and insulin insensitivity.

## Methods

### Reagents and materials

DMEM was purchased from HyClone; FBS was from Atlanta Biologicals; glutamine and penicillin-streptomycin (Penn-strep) were purchased from Invitrogen; DMSO, U0126, Rapamycin, LAMP1 (SC-19992) and LAMP2 (SC-18822) antibodies were purchased from Sigma-Aldrich. MK2206 was obtained from Selleckchem. Plasmids Tag5Amyc-GSK3β WT (Plasmid #16260) and Tag5Amyc-GSK3β CA (Plasmid #16261) were purchased from Addgene. Anti-GAPDH was purchased from Millipore (MAB374). Anti-TSC2 (for immunoblot) was from Abcam (Ab32554). Anti-puromycin was from Kerafast (EQ0001). LY294002 (9901), siRNA of ERK1/2 (6560), antibodies to P-ERK (9101), T-ERK (9102), P-Akt (4060), T-Akt (9272), P-TSC2 (3615), T-TSC2 (IF, 4308), P-S6K1(9234 S), T-S6K1 (9202 S), P-GSK3β (9336 S), T-GSK3β (12456 S), P-S6 (2215 S), T-S6 (2217 S) were purchased from Cell Signaling.

### Cell culture and treatment

HEK-293, MEFs and MCF7 cells were grown in DMEM (1:1) supplemented with 10% heat-inactivated FBS, 2 mM L-glutamine, 100 U/mL Penn-strep. For the starvation and insulin stimulation experiments, cells were grown in serum-starved DMEM media for overnight (16 h) prior to stimulation with 1 μM insulin for 15 min. For the pharmacological inhibitions, 16 h serum-starved cells were treated 2 h with DMSO (as a vehicle) or ERK inhibitor U0126 (10 μM), or Akt inhibitor MK2206 (10 μM) or PI3K inhibitor LY294002 (50 μM) or mTORC1 inhibitor rapamycin (300 nM) prior to stimulation with 1 μM insulin for 15 min. For knockdown of ERK1/2, cells were transfected with siRNA of ERK1/2 for 56 h prior to 16 h serum starvation, followed by stimulation with 1 μM insulin for 15 min. Cells were transfected with siRNA of ERK1/2 using the jetPRIME™ siRNA Transfection Reagent (Polyplus transfection).

### Immunoblot assay

Cells were rinsed with ice-cold PBS and harvested and lysed using RIPA buffer (50 mM Tris-HCl, ph 7.4, 1% NP40, 0.5% Na-deoxycholate, 0.1% SDS, 150 mM NaCl, 2 mM EDTA, and 50 mM NaF) including a cocktail of protease (Roche, Basel, Switzerland) and phosphatase (MilliporeSigma, Billerica, MA) inhibitors. Cells lysates were used for protein concentration measurement with the bicinchoninic acid (BCA) protein assay kit (Pierce, Rockford, IL), using BSA as standard. Lysates were then separated via SDS-PAGE followed by transfer to polyvinyl difluoride (PVDF) membranes. Blots were incubated in blocking buffer (5%, w/v, dried skimmed milk in Tris-buffered saline, pH 7.4, and 0.2% Tween 20, TBST) followed by overnight incubation with appropriate antibodies diluted in fresh blocking buffer. Blots were then washed three times with 1X TBST and then exposed to HRP-conjugated secondary antibodies diluted in blocking buffer for 75 min at room temperature and washed again. Blots were washed three times again prior to detection using appropriate image-developer.

### ERK and GSK3β co-immunoprecipitation analysis

HEK cells were seeded on 10 cm dish and cultured for 24 hours prior to transfection (Lipofectamine 2000, Invitrogen) with 12 µg of Myc-GSK3β. After 48 hours, the cells were lysed with NEMT buffer (50 mM Tris pH 7.5, 0.5% NP-40, 150 mM NaCl, and 1 mM EDTA with phosphatase inhibitor, Xpert phosphatase inhibitor cocktail (Gendepot) and Xpert protease cocktail (Gendepot)). The lysates were centrifuged at 13,000 g for 20 min and then the lysate was divided into 2 equal volumes. Cell lysates were immunoprecipitated with either ERK or IgG (as control) and immunoblotted with antibodies to ERK or GSK3β.

### P32 *in vitro* kinase assay

500 ng of recombinant substrate (GSK3β, SignalChem; MSK1, SignalChem) was combined with 100 ng of active kinase (ERK2, SignalChem) and then incubated in kinase buffer (25 mM MOPS, 25 mM MgCl_2_, 5 mM EGTA, 2 mM EDTA, 0.1 mg/mL BSA, 0.2 mM dithiothreitol (DTT)) with phosphatase inhibitor (Roche) 25 µM cold ATP (Invitrogen), and 1.2 µL of 0.250 mCi ^32^P ATP (PerkinElmer) for 1 hour at 30 °C. Addition of NuPAGE LDS buffer and sample reducing agent (Invitrogen) followed by boiling for 15 min terminated the kinase reaction. The samples ran on NuPAGE 4–12% Bis-Tris Gel (Invitrogen) followed by exposure to x-ray film (GE) for 1 hour.

### Confocal microscopy assay

Cells were grown on glass coverslips in 24-well plates overnight prior to treatment. After the end of the treatment, cells were rinsed with PBS and fixed with 4% paraformaldehyde (in PBS) at room temperature (RT) for 15 min. Cells were then rinsed five times with PBS prior to permeabilization with 0.1% Triton X-100 in PBS for 5 min. Cells were then rinsed five times with PBS and blocked with blocking reagent (0.1% saponin, 10% goat serum in PBS) for 90 min at RT. After blocking, cells were washed twice with PBS, followed by incubation with primary antibody in blocking reagent for overnight at 4 °C. Cells were then washed five times with PBS and incubated with labeled secondary antibodies for 1 h at RT in the dark. Cells were then washed five times with PBS and coverslips were then mounted with vectashield containing DAPI (H-1200) prior to microscopy. Images were acquired through 63x oil immersion objectives with either a Zeiss Axiotome fluorescence microscope with Apotome feature engaged or a Zeiss 710 confocal laser microscope (Zeiss, Oberkochen, Germany). Representative images are shown in all figures at the same exposure and magnification and in merged color images; colocalization is indicated by yellow and orange regions. For quantitative analyses, thresholded Pearson’s correlation coefficients were analyzed using raw images of split red and green channels. For each condition, images of at least 30–50 cells were analyzed.

### Measurement of Protein synthesis by SUnSET- puromycin end-labeling assay

After the end of the treatment, cells were incubated with puromycin (1 µM) for 30 min prior to cell lysis. Cell lysates were then subjected to immunoblot analysis. Anti-puromycin antibody was used to detect the level of puromycin incorporation. Protein synthesis was determined by measuring total lane signal from 250–10 kDa and subtracting unlabelled protein control. The signal from total protein loading was detected by coomassie staining as a control. Puromycin signal was normalized with the coomassie signal from each lane. Signals were quantified using ImageJ.

### Statistical analyses

All quantitative analyses are reported as mean ± SEM, unless otherwise specified. Statistical differences between groups were determined using ANOVA with Tukey’s post-hoc test. All statistical analyses were performed in Origin Pro (OriginLab Corporation, Northhampton, MA) with a significance level of *p < 0.05, **p < 0.01, ***p < 0.001, ^#^p < 0.05 and ^$^p < 0.01.
